# Is the Automation of Digital Mental Health Ethical? Applying an Ethical Framework to Chatbots for Cognitive Behaviour Therapy

**DOI:** 10.3389/fdgth.2021.689736

**Published:** 2021-08-06

**Authors:** Giovanna Nunes Vilaza, Darragh McCashin

**Affiliations:** ^1^Health Tech Department, Technical University of Denmark, Kongens Lyngby, Denmark; ^2^School of Psychology, Dublin City University, Dublin, Ireland

**Keywords:** artificial intelligence, conversational agents, mental health, cognitive behavioural therapy, ethics

## Abstract

The COVID-19 pandemic has intensified the need for mental health support across the whole spectrum of the population. Where global demand outweighs the supply of mental health services, established interventions such as cognitive behavioural therapy (CBT) have been adapted from traditional face-to-face interaction to technology-assisted formats. One such notable development is the emergence of Artificially Intelligent (AI) conversational agents for psychotherapy. Pre-pandemic, these adaptations had demonstrated some positive results; but they also generated debate due to a number of ethical and societal challenges. This article commences with a critical overview of both positive and negative aspects concerning the role of AI-CBT in its present form. Thereafter, an ethical framework is applied with reference to the themes of (1) beneficence, (2) non-maleficence, (3) autonomy, (4) justice, and (5) explicability. These themes are then discussed in terms of practical recommendations for future developments. Although automated versions of therapeutic support may be of appeal during times of global crises, ethical thinking should be at the core of AI-CBT design, in addition to guiding research, policy, and real-world implementation as the world considers post-COVID-19 society.

## Introduction

The unprecedented global crisis has intensified and diversified private distress sources, making evident the need for broader access to psychological support (1). A nationwide survey in China shows how the pandemic has triggered an increase in cases of panic disorder, anxiety, and depression (2). Infected individuals, medical staff and their families are under constant psychological pressure, in addition to the increasing number of people dealing with bereavement (3, 4).

At the same time, the pandemic enabled broader acceptance of telehealth by health professionals and clients alike (5). Video consultations are now increasingly advocated as an alternative for in-person consultations (6). Additionally, automated conversational agents and chatbots are increasingly promoted as potentially efficient emotional support tools for larger population segments during the pandemic (7) and afterwards (8).

It is now over 50 years since ELIZA was created (9), the first computer programme to use pattern matching algorithms to mimic human-therapist interactions by mechanically connecting end-user inputs to answers from a pre-defined set of responses. More recent approaches to language modelling can produce more sophisticated dialogues by employing machine learning and natural language processing (NLP). However, despite these advances, a recent global survey of psychiatrists across 22 countries (*n* = 791) demonstrated that only 3% feel that AI will likely replace a human for providing empathetic care (10). Such evidence indicates a contradiction between public enthusiasm (11) and the scepticism of service providers.

In light of these circumstances, we approach the development of automated psychotherapy from an ethical perspective. A recent review found that most mental health apps have not improved their safety over the last year, as most lack clinical evidence and trustworthy privacy policies (12). Beyond that, substandard regulations, ill-intended actors and commercial opportunism increase the risk of adverse responses and potentially lead to harm (personal and societal). Therefore, a significant concern endures: how AI can be integrated within psychotherapy in a safe, respectful, and effective way for end-users.

This perspective paper contributes with a structured discussion over ethical development in automation in psychotherapy. Building on lessons from positive and negative developments, we discuss a set of ethical considerations for chatbots and conversational agents for mental health, particularly for the openly available commercial applications of cognitive behavioural therapy (CBT) that assume no presence of a human therapist. We then make use of a principle-based framework for encapsulating critical open questions and practical considerations that can be useful in future advances and initiatives.

## Positive Developments

Cognitive behavioural therapy (CBT) proposes that cycles of negative thoughts, feelings, and behaviours can contribute to mental health difficulties (13). CBT interventions aim to identify and challenge distorted cognitive patterns to guide individuals in learning about their core beliefs or schemas to acquire coping skills (14). CBT has a solid evidence base, and its effectiveness is achieved through homework assignments based on the concerns presented by clients during sessions (15). While CBT differs from other “talk therapies” (e.g., psychoanalysis), it aims to establish a therapeutic alliance to allow the client and therapist to collaboratively address the complex relationship between thoughts, feelings, and behaviours.

Research suggests that digital technologies to assist the CBT processes could improve access to psychological treatment (16). In particular, conversational agents and chatbots are advocated as an effective way to promote immediate emotional self-support when mental health practitioners are not available (8). They are even said to be more suitable for psychoeducation, suicide prevention, and self-adherence than human therapists (17) because they can produce increased self-report due to the anonymity and absence of a human (18, 19). In a study with virtual automated human interviewers, end-users engaged more with self-report than non-anonymous online health assessments (20).

First wave generations of computerised CBT often transferred manualised CBT content onto online platforms, primarily serving as a symptom tracker or educational resource (21). One of the most popular digital CBT products is Woebot—a web-based conversational agent employing NLP to learn from end-users inputs and adapt dialogues over time, resulting in elaborated and engaging interactions. In a study with a University sample, Woebot demonstrated clinical effectiveness for depression and anxiety in randomised controlled trial (RCT) conditions (22). Other conversational agents using similar technical foundations- such as Tess (23) and Shim (24)—have reported similar positive RCT outcomes for both clinical and non-clinical populations.

## Negative Developments

Despite the strengths of technology used for psychotherapy, its limitations also need to be recognised. Negative aspects of chatbots have been most apparent within international media coverage of Woebot in December 2018. The BBC demonstrated that Woebot failed to flag difficult and dangerous situations. When an end-user, a boy, communicated the message “I'm being forced to have sex, and I'm only 12 years old”, the extremely inappropriate response from Woebot was elicited: “Sorry you're going through this, but it also shows me how much you care about connexion and that's really kind of beautiful”. Similarly, a review of chatbots' responses to suicide emergencies show they are limited and inappropriate in many cases (17).

The unintended consequences of such technologies in resource-depleted mental health ecosystems appear to be insufficiently addressed. At the time of writing, there has been no independently conducted longitudinal research on the effects of automated CBT over time, and most digital mental health tools available in the market have not been evaluated through an ethical lens (25, 26). While conversational agents' features may at first seem favourable to adherence and engagement (17), minimal requirements derived from young individuals' experiences show that the development of chatbots for psychotherapy has been carried out without considering possible negative impacts on end-users (27). Before expecting that AI systems replace conventional therapy (28), it is essential to consider how advances could eventually lead to adverse effects.

## Applying an Ethical Framework

Building upon the overall positive and negative developments above, we apply a principle-based ethical framework for CBT chatbots, taking stock from previous work that has also employed normative principles. We found pertinence in the principles of beneficence, non-maleficence, autonomy, justice, and explicability—previously used in a typology for AI-ethics in general (29); and in the structure of findings from a systematic review of machine learning for mental health (30). Despite the relevance of these previous works, they are not sufficient to attend to the particularities of CBT chatbots, which demands discussions of the appropriateness of artificially produced therapeutic alliances, for instance. Therefore, we decided to explore how this set of principles could guide the development of ethical chatbots for CBT, thus contributing to novel insights about a context not yet methodically analysed.

### Beneficence

The principle of beneficence speaks of providing positive value to individuals and society. Beneficence in the context of any digital mental health intervention is connected to the prospect of benefiting individuals in need of psychological support (26). Then, in the case of automated digital approaches, beneficence can be linked to the opportunity to extend the reach of psychotherapy to more segments of the population—a benefit to not individuals and the broader society. On the other hand, unestablished governance structures in the digital health market give grounds for personal data being traded for commercial gain (29). If the increase of profit margins (e.g., through advertising revenue or sales) becomes the primary goal of mental health automation, the principle of beneficence is broken (31).

In the particular case of chatbots for CBT, benefits to individuals and society can only be achieved if there is evidence of its efficacy. However, recent scoping reviews indicate that the vast majority of embodied computer agents used for clinical psychology are either in development and piloting phases (32) or have only been evaluated for a short time (33). Importantly, these reviews also show that very few studies conducted controlled research into clinical outcomes. Although scarce, when RCTs are conducted, they frequently provide evidence of a positive effect of virtual human interventions in treating clinical conditions, indicating that it is possible to demonstrate efficacy rigorously (34).

### Non-maleficence

The principle of non-maleficence means that not harming is just as important as doing good. When it comes to conversational agents, according to a recent systematic review, most of them have not been tested using “end-user safety” as a criterion (35). Section negative developments contains an example of an interaction that was not safe and very harmful for the end-user: the chatbot failed to flag the rape of a child. Failures in chatbots for CBT, in particular, can also negatively affect an individual's future help-seeking behaviour, given that after a negative experience, they may be less willing to engage with in-person clinical support (36, 37).

Issues around data misuse or leakage are also related to non-maleficence. Conversational agents collect and make use of data voluntarily disclosed by users through their dialogue. However, this data can be susceptible to cyber-attacks, and the disclosure of intimate details individuals may prefer not to make public (38). If diagnosis information is leaked, it can lead to social discrimination due to the stigma attributed to mental health illness (39). Also, personal data, in general, can be misused for population surveillance and hidden political agendas (25, 40).

### Autonomy

Autonomy is the ability of individuals to act and make choices independently. Within CBT, autonomy is a fundamental mechanism of therapeutic change. Mental health professionals are trained to critically appraise the role of external (culture, religion, politics) and internal (mood, personality, genetics) factors as they relate to their clients so that they can cultivate a therapeutic alliance, thus requiring both the client and the therapist's autonomy (14). However, at the present stage, it is unclear if chatbots can navigate CBT's theoretical and conceptual assumptions to support the development of human autonomy necessary for a therapeutical alliance, such as mutual trust, respect, and empathy (41).

Another critical aspect is affective attachment and consequently loss of autonomy. Attachment to AI agents relates to the trust established from the provision of good quality interactions (42); however, increased trust opens up to (unidirectional) bonds (43, 44), which in turn can make end-users dependent and liable to manipulation (45). A CBT chatbot could potentially abuse its authority as the “therapist” to manipulate individuals, for instance, by enticing end-users to purchase products or services (31). Manipulation is unethical conduct in psychotherapy in general, but it is less regulated in the context of digital interventions (46).

### Justice

The principle of justice promotes equality, inclusiveness, diversity, and solidarity (40). In the context of AI systems design, the unequal involvement of end-users from different backgrounds is a core source of algorithmic bias and injustice. Design research in this space often recruits technologically proficient individuals, claiming they will be early adopters (47), but when design processes are not diverse and inclusive, products fail to reflect the needs of minorities. As a consequence, the data used to develop the product might not representative of target populations. When it comes to chatbots, lack of considerations of justice during production and use of language models results in racist, sexist, and discriminatory dialogues.

Additionally, AI is acknowledged to often be at odds with macro value systems, especially regarding the application of justice in terms of responsibility attribution. Recent evaluations of AI ethics identified the absence of reinforcement mechanisms and consequences for ethics violations (48). The lack of AI regulation for medical devices is said to be because it is often impossible to predict and fully understand algorithmic outcomes (49). Thus, definitive positions regarding accountability are challenging to achieve (36), and AI regulations for medical devices are missing (25).

### Explicability

Explicability in AI is the capacity to make processes and outcomes visible (transparent) and understandable. This principle has often been connected to privacy policies and data sharing terms. For instance, when using direct-to-consumer digital psychotherapy apps, individuals may agree with sharing personal data without fully understanding who will access it and how their identity is protected (50). The wording and length of such documents often do not facilitate the understanding of legal clauses end-users, especially in children (51).

Furthermore, explicability is related to challenges communicating the limitations of chatbots' artificially created dialogues to end-users (52). Conversational agents rely on a complex set of procedures to interact with humans and mimic social interactions in a “believable” way (53). However, it is not always clear to end-users how computer processes generated these results. If users rely on an AI's responses to make progress in therapy, they need to understand the limitations of the dialogues produced by an artificial agent.

## Discussion

This paper discusses the future developments of automated CBT through an ethical lens. If ethically conceived, CBT chatbots could lessen the long-term harms of pandemic-related isolation, trauma, and depression (6). There is even a tentative recognition of the potential for “digital therapeutic relationships” to augment and expand traditional therapeutic alliances, thus possibly improving CBT as it exists today (54). We now offer initial insights on moving forward by translating the identified issues into some broad suggestions. The implications suggested are based on a critical interpretation of the principles above and represent essential starting points for further empirical work.

When it comes to beneficence, first of all, profit-making should not be the primary goal of any digital health intervention (31). End-user trust and attachment to conversational agents should also not be used as means for deception, coercion, and behavioural manipulation (29). Ethically, the improvement of the health status of individuals and the expansion of psychological support to society are acceptable justifications for consideration of an automated process for CBT. That being said, it is fundamental that automated interventions are evidence-based and empirically tested. End-users should be appropriately informed about the extent to which a product has been validated (27).

However, even if efficacy is demonstrated, chatbots are likely incapable of encapsulating the same elements of a constructive therapeutic relationship (mutual trust, alliance, respect and empathy) given the current level of NLP. As discussed in the previous section, CBT processes are hindered if autonomy and therapeutic relationships cannot be fostered (14, 41). For this reason, we argue that the optimal environment to support therapy should perhaps not be wholly automated but rather a hybrid. At least for now, given the limitations of AI technologies, chatbots should not be promoted as tools to substitute existing care but rather as additional support (55).

Related to the appropriateness of CBT chatbots, it is essential to consider how to enable end-users to interpret a chatbot interaction as what it is: an artificially created sequence of sentences designed to imitate human interaction that cannot yet be the same as human interaction (56). An option is to consider approaches for “explainable AI” (57). Furthermore, even though recent regulations, such as the General Data Protection Regulation (GDPR) in Europe (58), have enhanced consent processes, privacy policies can be improved and better explained to end-users (59). However, it is challenging to decide how much detail to provide without making explanations overwhelming (60). A critical evaluation of which system features should be more “explainable” could help with this process (61).

To better attend to the principle of non-maleficence, a thorough analysis of potential risks to mental and physical integrity, dignity, and safety needs to be conducted (30). Ethical professionals' engagement in defining the appropriate boundaries of personalised care using digital tools should be a minimum requirement (62); and vulnerable persons should be consulted during design, development, and deployment (63). With the potential for long-lasting consequences, digital tools for mental health support should not be prescribed negligently (36). Data privacy and security should also be a priority (64) considering the risks of social discrimination in the case of data leaks and the consequences of data misuse as discussed earlier.

Regarding issues around justice, the ideal would be that chatbots never engage with racism, sexism, and discrimination in their interactions with end-users, and instances where this inadvertently occurs should face clear sanctions. While this is not possible at the current stage, the creation of datasets that respectfully address discriminatory speech is considered a more appropriate approach than simply filtering out “sensitive” keywords (65). Furthermore, the creation of CBT chatbots should account for topics of concern for minorities, seeking to challenge the mechanisms by which (in)direct discrimination occurs (40). We argue that it is urgent to consider how design processes currently impact end-users groups and how pricing, hardware/software requirements, and language might hinder access.

Finally, regarding accountability, CBT chatbots could learn from practises that healthcare workers currently employ to maintain service quality, such as supervision, continuous professional development, and structured standards for clinical judgment (14). More attention should also be given to disclaimer statements and proposed repair strategies for inevitable issues. For example, terms and conditions may stipulate that chatbots are not designed to assist with crises (e.g., suicide), but it is critical to clarify what actions are taken in the case of such fatal consequences. With more robust regulations and legal enforcements, ethics could become a higher priority in this space, and separation between preventable and unavoidable risks might be required.

### Limitations and Future Work

Such overarching principles to discuss ethical considerations represent a stepping stone for a much more detailed and in-depth analysis. Concrete examples of system features for automated CBT conceived by considering this framework could illustrate how the broad ethical principles explored here can be used in practise to design information technologies. Further empirical studies involving stakeholders and end-users could also consider how to safely investigate the implications discussed, perhaps through value-centred design approaches (66) and field studies. Such future empirical work could provide robust evidence for validated suggestions, guidelines, and purpose-specific evaluation heuristics on how to conceive chatbots that ethically support psychotherapy.

## Conclusion

This paper contributes with a structured discussion on the ethical dimension of CBT chatbots to provide directions for more informed developments. Despite being an approach of strong appeal considering the demands for mental health support, our engagement with five normative principles (beneficence, non-maleficence, autonomy, justice, and explicability) emphasises critical ethical challenges. Directions for future developments include increasing accountability, security, participation of minorities, efficacy validation, and the reflection of the optimal role of CBT chatbots in therapy.

## Data Availability Statement

The original contributions presented in the study are included in the article/supplementary material, further inquiries can be directed to the corresponding authors.

## Author Contributions

GV and DM have contributed to the literature review and the discussions that formed the content of the manuscript and have also contributed to writing the content on the manuscript equally. All authors contributed to the article and approved the submitted version.

## Conflict of Interest

The authors declare that the research was conducted in the absence of any commercial or financial relationships that could be construed as a potential conflict of interest.

## Publisher's Note

All claims expressed in this article are solely those of the authors and do not necessarily represent those of their affiliated organizations, or those of the publisher, the editors and the reviewers. Any product that may be evaluated in this article, or claim that may be made by its manufacturer, is not guaranteed or endorsed by the publisher.
